# Japanese Food Data Challenge the Claimed Link between Fukushima’s Releases and Recently Observed Thyroid Cancer Increase in Japan

**DOI:** 10.1038/s41598-017-10584-8

**Published:** 2017-09-06

**Authors:** Georg Steinhauser, Manuel Chávez-Ortega, Jan-Willem Vahlbruch

**Affiliations:** Leibniz Universität Hannover, Institute of Radioecology and Radiation Protection, Herrenhäuser Str. 2, 30419 Hannover, Germany

## Abstract

Internal, high-dose exposure with radioiodine is known to increase the risk for thyroid cancer in children and adolescents. Ingestion of contaminated food is generally regarded a dominant route of internal exposure. We analyzed the huge data set of the post-Fukushima food monitoring campaign and deployed a conservative model for the estimation of the doses to the general public in a worst-case scenario. Our data suggest that the committed equivalent ingestion doses to the thyroids of the affected Japanese public, even in the utmost conservative approach, remained below the limit on ingestion of radioiodine in foodstuffs and beverages of 50 mSv (as thyroid equivalent dose). This level of 50 mSv is also the intervention level for the administration of stable iodine, mainly after inhalation. Our study hence suggests that, based on the food data, the internal exposure of Japanese residents was too low to cause a statistically discernible increase in thyroid cancer, even if the contribution from inhalation is taken into account. The data also indicate that the governmental efforts in the food monitoring campaign were successful and cut the thyroid doses to the public by a factor of approximately 3 compared to a scenario without any monitoring.

## Introduction

Releases of radionuclides from the crippled Fukushima Daiichi nuclear power plant (FDNPP) in 2011 triggered public concern for their effects on environmental and human health. Much concern was expressed about food safety in the public^1^. Lessons from the Chernobyl nuclear accident^2^ (1986) include that incorporated radioiodine, especially ^131^I with its short half-life of only 8.03 days, causes significant doses to the thyroid. Epidemiological studies have shown that more than 7000 thyroid cancer cases in children and adolescents had to be attributed to the releases from Chernobyl^3, 4^ and the lack of professional response to this accident that would have been desirable: rapid evacuation, food safety campaigns, and a far-reaching stable iodine prophylaxis for the affected population. The situation after the Fukushima nuclear accident, however, was completely different. On the one hand, Fukushima’s radioiodine releases (about 150–200 PBq ^131^I) accounted to about 10% of the releases from Chernobyl^5^, and more than half of this amount was blown offshore and deposited in the Pacific Ocean^6–8^, thus causing relatively moderate dose rates on the Japanese mainland^9^, at least outside the evacuation zone^10^. On the other hand, Japanese authorities reacted quickly and initiated a timely evacuation, launched an unprecedented food monitoring campaign^11, 12^, and distributed, where necessary, stable iodine drugs to reduce ^131^I intake and thyroid doses. Since the evacuation had been completed in a timely fashion, none of the distributed iodine drugs had to be taken by the evacuees, but only by the workers on the FDNPP site^13, 14^.

The Fukushima nuclear accident may cause additional cancer cases, especially amongst workers who received effective doses of 100 mSv or more^15^. For the Japanese public, even the most optimistic estimates expected some additional cases of cancer^16^. However, due to the characteristics of the Fukushima accident and the great efforts in the mitigation of its health consequences, most scholars did not expect this increase in cancer cases amongst members of the public or even to the highly-exposed workers to be statistically discernible from the background rate of cancer^16, 17^. Nevertheless, the United Nations Committee on the Effects of Atomic Radiation (UNSCEAR), in a conservative approach, did not rule out the possibility for an increased thyroid cancer risk amongst infants and children^13^. However, previous studies suggested much lower doses to the thyroids of (young) residents than what would be expected to visibly increase thyroid cancer risk^18–21^. Therefore, most scientists within the radiological community were puzzled, when a drastic increase in thyroid cancer in young Japanese was reported as early as January 2015, claiming a link to the radioactive releases from FDNPP^22^. This study was viewed controversially by many and intensely debated^23–32^. The most pronounced point of criticism was that the epidemiological data were not (or just weakly) linked to actual exposure of the affected population with radioiodine. Further is important to note that diagnoses of thyroid cancer are globally on the rise, but not necessarily because of an increasing cancer incidence but mainly because of an increasing detection by improved diagnostic means^33, 34^.

After a nuclear accident, radionuclides are primarily incorporated by inhalation and ingestion of contaminated food, where the latter is generally regarded as the dominant source of incorporation^9, 35, 36^, although some studies suggested that, for the case of Fukushima, inhalation may be somewhat more dose-relevant than in case of Chernobyl due to rigorous milk restrictions in Japan^18, 37^. Studies on the actual internal exposure from radioiodine in residents of affected areas using whole-body counting (WBC) were naturally limited in numbers of participants, mainly as a consequence of the short half-life of ^131^I^36^. Several studies agreed that for the actual assessment of exposure of the thyroid, not enough data are at hand and that estimates of the thyroid exposure involve a high uncertainty^20, 38^.

Although a lot of work has been done in assessing the incorporation of radionuclides by WBC and estimates on the doses through inhalation, internal exposure with ^131^I through contaminated food was not studied nearly as comprehensively. Japanese authorities under the lead of Ministry of Health, Labor and Welfare (MHLW) launched an unprecedented food monitoring campaign by measuring hundreds of thousands of food samples. The radioiodine fraction of the data published in that campaign^39^ (see Table S1) are used herein to study contamination levels and trends as well as to derive dose estimates following ingestion of contaminated foods. Since contaminated food has the potential to affect a much higher fraction of the population than sole exposure from inhalation, this analysis allows a much more comprehensive risk assessment for thyroid cancer as a consequence of the Fukushima nuclear accident than sole estimates on the inhalation of contaminated air. Inhalation of radioiodine by air may cause different exposure depending on the different absorption of the various iodine species (e.g., particulate, gaseous or organically bound iodine). The ICRP’s dose conversion factors take this different chemical behavior into account. For the present estimate of the internal exposure through ingestion of contaminated foods, it is important to note that such estimate is only possible by analyzing food data from the governmental monitoring campaign. Privately grown or collected foods, as outlined previously by Hayano *et al*.^40^, pose the risk of a massive exposure by incidentally bypassing the governmental safety measures that have been installed for “regular” market foods. Fukushima prefecture, therefore, now offers radioanalytical facilities for the checkup of home-grown or privately collected foods.

## Results and Discussion

### Contamination Levels

For most food samples analyzed, the MHLW food database distinguishes between “pre-market” (samples taken directly at the producer or at a production facility before reaching the customers) and “post-market” (samples taken at shops and markets in competition with customers). A small fraction of samples was not categorized in one of the two categories (“not specified”). For the discussion herein, any “not specified” samples in the database were counted as “post-market” samples, which should represent the utmost conservative approach for the dose estimates. The basic food safety characteristics of the food monitoring campaign (^131^I only) are summarized in Table 1. The main food categories relevant for ^131^I monitoring were above-ground vegetables, cattle milk, fishery products, mushrooms, fruits and berries, algae, meat and eggs, below-ground vegetables, and tea (see Table S2).

**Table 1:** Basic characteristics of the MHLW food data for ^131^I in food in the most relevant food categories. Data are split into pre-market samples and post-market samples in order to illustrate the effectiveness of the campaign. For a consistent, conservative approach, food data with a “not specified” market category were counted as post-market samples.

	Above-ground vegetables			Cattle milk & dairy products			Fishery products		Mushrooms	
	pre-market	post-market/ not specified		pre-market		post-market/ not specified	pre-market	post-market/ not specified	pre-market	post-market/ not specified
First measurement	2011-03-17	2011-03-18		2011-03-16		2011-03-19	2011-03-23	2011-03-20	2011-03-25	2011-03-23
First detection	2011-03-17	2011-03-19		2011-03-16		2011-03-19	2011-03-24	2011-03-30	2011-03-25	2011-04-01
Last detection	2011-05-23	2011-06-07		2011-03-24		2011-05-06	2011-04-18	2011-06-13	2011-04-14	2011-05-12
First exceedance	2011-03-18	2011-03-19		2011-03-16		2011-03-19	N/A	2011-04-01	2011-04-14	2011-04-01
Last exceedance	2011-03-30	2011-04-11		2011-03-22		2011-03-19	N/A	2011-04-18	2011-04-14	2011-04-08
No. of measurements between first measurement and last detection	575	2179		66		259	17	593	51	191
No. of measurements between first and last detection	575	2172		66		259	16	592	51	190
No. of detectables	361	582		60		147	11	127	32	68
No. of exceedances	88	14		14		9	0	4	1	2
Percentage of exceedances in measurements until last detection	15.3%	0.6%		21.2%		3.5%	0.0%	0.7%	2.0%	1.0%
Highest ^131^I activity concentration (Bq/kg)	54100	12000		5300		5200	103	12000	3500	12000
	**Fruits & berries**		**Algae**		**Meats & eggs**		**Below-ground vegetables**		**Tea**	
	**pre-market**	**post-market/ not specified**	**pre-market**	**post-market/ not specified**	**pre-market**	**post-market/ not specified**	**pre-market**	**post-market/ not specified**	**pre-market**	**post-market/ not specified**
First measurement	2011-03-19	2011-03-18	N/A	2011-03-24	2011-03-20	2011-03-15	2011-03-19	2011-03-19	2011-05-06	2011-05-02
First detection	2011-03-19	2011-03-19	N/A	2011-03-24	2011-03-26	2011-03-28	2011-03-19	2011-04-03	2011-05-07	2011-05-02
Last detection	2011-03-25	2011-05-04	N/A	2011-07-15	2011-03-27	2011-04-08	2011-03-28	2011-05-02	2011-05-24	2011-05-10
First exceedance	N/A	N/A	N/A	2011-05-21	N/A	N/A	N/A	N/A	N/A	N/A
Last exceedance	N/A	N/A	N/A	2011-05-21	N/A	N/A	N/A	N/A	N/A	N/A
No. of measurements between first measurement and last detection	21	82	None	29	14	44	5	33	51	12
No. of measurements between first and last detection	21	81	N/A	29	7	42	5	22	50	12
No. of detectables	21	22	N/A	22	7	12	4	8	2	7
No. of exceedances	0	0	N/A	1	0	0	0	0	0	0
Percentage of exceedances in measurements until last detection	0.0%	0.0%	N/A	3.4%	0.0%	0.0%	0.0%	0.0%	0.0%	0.0%
Highest ^131^I activity concentration (Bq/kg)	1400	300	N/A	2200	45	19	990	1000	6.75	2.34

**Figure 1:**
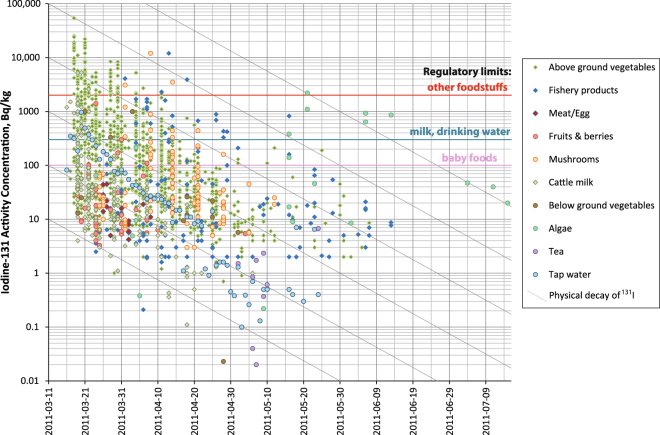
Activity concentrations (Bq·kg^−1^) of ^131^I in food over time (at the date of sampling). Regulatory limits were 100 Bq·kg^−1^ for baby foods, 300 Bq·kg^−1^ for milk and drinking water, and 2000 Bq·kg^−1^ for vegetables (except corms, tubers and roots) and aquatic products. Diagonal gray lines indicate the physical decay behavior of ^131^I according to its half-life. Please note that only food items with detectable ^131^I activity concentrations are displayed.

Although occasionally high activity concentrations of ^131^I were found in the early aftermath of the Fukushima nuclear accident, the actual number of food samples exceeding the Japanese regulatory limits was relatively small (Fig. 1). Figure 1 also illustrates the decline characteristics of ^131^I in food: the activities decrease faster than due to physical decay alone (as shown by the gray, diagonal decay lines) in almost all food categories studied herein. Only algae exhibited rather constant activity concentrations, as they compensate for physical decay by hyperaccumulation of ^131^I for several weeks^41^.

The food safety campaign started on March 16, 2011; *i.e*. one day after the major releases from FDNPP. The maximum activity concentration was found in spinach from Ibaraki prefecture south of Fukushima prefecture on March 18, 2011 (54,100 Bq·kg^−1^, pre-market). Although 15.3% of all pre-market vegetable samples exceeded the regulatory limit for ^131^I (between the first measurement and the last detection of ^131^I in the specific food category as shown in Table 1), only 0.6% of the samples in the post-market category exceeded the regulatory limit. This indicates both a high efficiency of the monitoring campaign in terms of preventing highly contaminated foods from reaching the customers and a high overall degree of food safety^42^.

Milk and dairy products contaminated with ^131^I are the most sensitive food items with respect to their crucial role as the dominant thyroid dose contributors in children and adolescents. The first detection in cattle milk was observed right on March 16 and was as high as 1190 Bq·kg^−1^ in Fukushima. The latest detection in cattle milk, in minute concentrations, was observed on May 6, 2011. However, only few detections were reported after April 15, 2011. Of 325 dairy samples measured until May 6, 23 samples (7.1%) exceeded the regulatory limit of 300 Bq·kg^−1^, and only 9 (3.5%) of the exceedances were potentially obtained in the post-market segment (all 9 were classified as “not specified”, none was explicitly obtained in the post-market). The maximum ^131^I activity concentration in dairy products was found in a “pre-market” milk sample from Fukushima prefecture on March 20 (5300 Bq·kg^−1^). For comparison, early limits after Chernobyl for ^131^I in milk in affected areas had been set to 1000 Bq·L^−1^.

Activity concentrations in tap water peaked on March 20, 2011 with 965 Bq·L^−1^ in Fukushima. Over the course of the accident, tap water exceeded the regulatory limit (300 Bq·L^−1^) on 5 days between 03/17 and 03/22. Fish and algae showed a different radioecological behavior than other types of food with a long activity increase period (indicating high accumulation) and late exceedances (Fig. 1). Nonetheless, only 0.7% of the fishery products in the post-market segment exceeded the regulatory limit.

In total, 4222 samples were measured and included in the MHLW database during the “^131^I period”, 800 of which were taken in the pre-market and 3422 were taken in the post-market or were not specified into one of the two categories. In the entire pre-market section, 103 samples (12.9%) exceeded the regulatory limits; in the post-market/not specified category, 30 samples (0.9%) exceeded the limits.

### Dose estimates

A conservative dose model was applied, in which we assumed that reference persons of all ages consumed exclusively the highest-contaminated foods available on the Japanese market. In this model, the activities from the highest-contaminated foods (of each category) that were available day by day were summed up; the types and amounts of foods for this calculation were chosen according to the composition of the typical Japanese food basket in all age groups (taken from^43^). It is unclear however, what the affected population actually ate (or was given to eat) in the early aftermath of the accident and evacuation.

**Figure 2:**
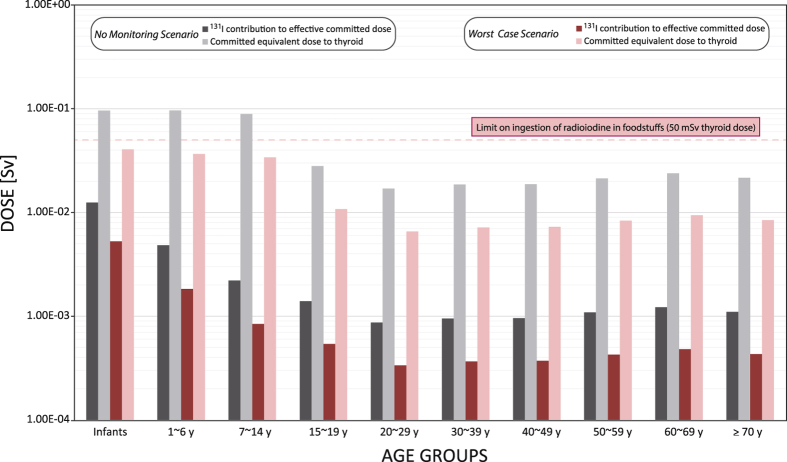
Dose contributions for various age groups caused by ^131^I in food for customers in Japan as calculated by conservative food intake assumptions. Two scenarios are compared: a highly conservative but potentially realistic worst-case scenario (pink bars) and, a no-monitoring scenario (gray bars), respectively, that reflects the causes if no food monitoring had been conducted in Japan after the accident. For each age group and each scenario, the equivalent dose to the thyroid is shown along with the contribution of ^131^I to the effective committed dose. The dotted line, for illustration, shows the limit on ingestion of radioiodine in foodstuffs and beverages (50 mSv).

In order to demonstrate the effectiveness of the monitoring, a further distinction in pre-market and post-market was made. First, for the “worst-case scenario”, only “post-market” and “not specified” foods were combined and used for the calculation. Second, a hypothetical scenario was designed for reasons of illustrating the effectiveness of the monitoring: in this scenario, also the pre-market samples were included into the dose calculation, in order to exemplify the effects for the public if no monitoring had taken place at all. Hence, for this scenario, which we called “no-monitoring scenario,” samples from all market categories (including those samples that were actually removed from the pre-market once they exceeded the regulatory limit) were included into the food basket. Thereby it is possible to show to which extent the food monitoring has actually reduced the exposure of Japanese citizens. Both scenarios are illustrated in Fig. 2.

In the approach illustrated in Fig. 2, no further distinction between male and female consumers was made because also the dose conversion factors do not distinguish between genders. In the Supporting Information, however, the dose calculations for each gender are given in Table S3, showing men receiving a slightly higher dose due to higher consumption rates.

The ICRP recommendation is that an intervention level of 500 mSv equivalent dose to the thyroid is ‘almost always justified’ for the administration of stable iodine in response to nuclear emergencies and that an optimized value should not be lower than 50 mSv^44^. This intervention level is mainly intended in response to inhalation of radioiodine species. The Nuclear Safety Committee in Japan also set a maximum dose of 50 mSv as thyroid equivalent dose for exposure from ingestion of contaminated food and beverages. Figure 2 shows that in the “worst-case scenario”, even for infants and young children, this level of 50 mSv was not reached. In the “no monitoring scenario,” the ‘optimized value’ was exceeded, whereas the ‘almost always justified’ tenfold intervention level was not nearly reached (ingestion dose only). The contribution of food-borne ^131^I to the effective committed dose, however, exceeded the recommended maximum additional effective committed dose of 1 mSv for the general public for children ≤6 years in our ultra-conservative scenario. It is obvious that our worst-case scenario is so conservative that, with all possible fluctuations of the contamination levels and single spikes that may have gone unnoticed in the monitoring campaign, any realistic exposure through the consumption of contaminated food is probably in the single digit or low double digit percent level of what is shown in Fig. 2. The conservative approach of our model is exemplified by the assumption of a complete transfer of radioiodine from the raw food into the final meal. One may assume that in the course of food preparation (e.g., peeling or cooking), a significant fraction of the contained radioiodine may be lost (either by mechanical removal or by dilution in the cooking water^45^, but not so much by heat-induced volatilization of the radioiodine species^46, 47^).

The dose estimates presented herein are in good agreement with previous studies: Tokonami *et al*.^18^ found that the equivalent doses to the thyroid due to ingestion in the measured persons were all below 40 mSv (most individuals received equivalent doses to the thyroid of less than 5 mSv). Equivalent thyroid doses due to inhalation were quite similar as all were below 35 mSv; again, most individuals in their study received less than 5 mSv. The dose estimates presented by UNSCEAR^13^ were also in the range of this study: Amongst the citizens of Fukushima prefecture that were not evacuated, the thyroid equivalent doses (from both inhalation and ingestion) ranged, in the first year, from 33–52 mGy for 1-year olds, 15–31 mGy for 10-year olds and 7.8–17 mGy for adults. Equivalent doses to the evacuated public, according to UNSCEAR ranged higher (47–83 mGy for 1-year olds; 27–58 mGy for 10-year olds; 16–35 mGy for adults; all for the first year)^13^. Most of this exposure occurred before and during the evacuation. These higher equivalent doses for the evacuated public suggests that, for these people, inhalation was the primary pathway for internal exposure. For people from Fukushima prefecture, but outside the evacuation zone, and especially for people from neighboring prefectures, however, ingestion remains the dominant route for internal exposure with radioiodine^13^.

With only few exceptions, the equivalent dose to the thyroid can be estimated to have remained below 100 mSv. It is important to note that the effects of such low doses are not yet sufficiently understood and that single thyroid cancer cases as a result of an exposure in the range of <100 mSv equivalent dose to the thyroid cannot be ruled out. However, based on the knowledge gathered over decades, there is no indication that would explain a statistically significant and visible increase of the thyroid cancer rate beyond its natural fluctuation uncertainty caused by such low doses.

The analysis of the food data suggests low exposure of the public due to ^131^I-contaminated foods in the early aftermath of the Fukushima nuclear accident. If the MHLW food monitoring data are representative for the Japanese food basket at the time of the accident, the analysis of these data suggests that the thyroid cancer cases observed after the Fukushima accident are unlikely to be linked to the releases of radioiodine from FDNPP, but are rather caused by a screening effect^23, 48^ or other confounding factors. The representativeness of the data set is primarily limited by the lack of coverage of privately produced/collected food. Although minor biases in the campaign, e.g. during sampling, cannot be ruled out, the sheer data volume in the database should render them insignificant. There is currently no indication to believe that the food data discussed herein should not represent the Japanese food basket. One possibly important condition for the accuracy of this study’s main conclusion is that the current physiological and epidemiological knowledge applies to the affected population. Genetic profiling is currently being undertaken for the cancer tissue. First results suggest a “different oncogenic profile” from the Chernobyl cancer cases^49^. Another limitation is that it remains unknown how the “triple disaster” of March 2011 (logistically) affected the composition of the food basket of the affected population.

The analysis of the food data presented herein also shows that the food monitoring efforts of the Japanese authorities were successful in mitigating the adverse effects of radioiodine intake, by cutting the doses down to 1/3 compared to a hypothetical scenario without any food monitoring.

## Methods

For the estimation of the internal exposure with radioiodine, a dose model was applied, giving a conservative but realistic estimate of the actual intake of activity with food (see Equation 1). For this dose estimate, the utmost conservative approach was chosen. Radiation damage not only depends on the intake of activity but also on age, because children are more vulnerable than adults or seniors. This is reflected by age-dependent dose conversion factors published by the International Commission on Radiological Protection (ICRP)^50^. Average Japanese food intake numbers for age groups 1–6; 7–14; 15–19; 20–29; 30–39; 40–49; 50–59; 60–69; and 70+ years, respectively, were taken from ref. 43. These individual intake numbers were multiplied with the highest ^131^I activity concentration of the respective food item in the MHLW database for each day. Finally, the days’ intake was summed up to represent the total intake of the entire food basket. In other words, the exposure was calculated as if the food basket of the reference person in our scenario exclusively consisted of the highest-contaminated food items available on that day in the entire Japanese market. The reference person in our scenario only drank tap water, even on days when it exceeded the regulatory limit.

1 $${{\rm{E}}}_{{\rm{ing}},{\rm{j}}}=\sum _{{\rm{n}}}{{\rm{p}}}_{{\rm{n}}}{{\rm{U}}}_{{\rm{n}},{\rm{j}}}\,\sum _{{\rm{r}}}{{\rm{C}}}_{{\rm{n}},{\rm{r}}}{{\rm{g}}}_{{\rm{ing}},{\rm{r}},{\rm{j}}}$$

In Equation (1), E_Ing,j_ stands for the effective dose [Sv] for the reference person j through ingestion of contaminated food; C_n,r_ is the activity concentration in Bq·kg^−1^ or Bq·L^−1^, respectively, of the radionuclide r in food item n. The fraction of local produce p_n_ in the reference person’s (j) food basket was assumed to be 1. U_n,j_ is the daily intake of a food item n in kg by reference person j, and g_Ing,r,j_ is the ingestion dose coefficient for radionuclide r and reference person j in Sv·Bq^−1^. The food categories of n are tap water, above ground vegetables, below ground vegetables, cattle milk, fishery products, meat/eggs, fruits/berries, mushrooms, algae, and tea.

Exposure for infants was calculated from the intake numbers of women of the age group 30–39 years, and a transfer factor for radioiodine of 0.6 from the mother’s intake to breast milk as outlined in German legislation^51^. This transfer factor is even more conservative than suggested by the ICRP (0.3)^52^. For the conservative approach, it was further assumed that the infants were exclusively breastfed.

## Electronic supplementary material

Table S1

Table S2

Table S3
